# A Phenolic Fraction from* Catharanthus roseus* L. Stems Decreases Glycemia and Stimulates Insulin Secretion

**DOI:** 10.1155/2018/7191035

**Published:** 2018-11-11

**Authors:** Janet Alejandra Espejel-Nava, Elisa Vega-Avila, Francisco Alarcon-Aguilar, Alejandra Contreras-Ramos, Guadalupe Díaz-Rosas, Gloria Trejo-Aguilar, Clara Ortega-Camarillo

**Affiliations:** ^1^Posgraduate Program in Experimental Biology, DCBS, Autonomous Metropolitan University, Av. San Rafael Atlixco 186, Col. Vicentina, CP 09340, Del. Iztapalapa, CDMX, Mexico; ^2^Experimental Hematology Laboratory, Department of Health Sciences, Autonomous Metropolitan University, Av. San Rafael Atlixco 186, Col. Vicentina, CP 09340, Del. Iztapalapa, CDMX, Mexico; ^3^Pharmacology Laboratory, Department of Health Sciences. Autonomous Metropolitan University, Av. San Rafael Atlixco 186, Col. Vicentina, CP 09340, Del. Iztapalapa, CDMX, Mexico; ^4^Laboratory of Developmental Biology Research and Experimental Teratogenicity, Children Hospital of Mexico Federico Gomez (HIMFG). Dr. Márquez No. 162, Col. Doctores, CP 06720, Delegación: Cuauhtémoc, CDMX, Mexico; ^5^Instrumental Analysis Laboratory, Department of Biotechnology, Autonomous Metropolitan University, Av. San Rafael Atlixco 186, Col. Vicentina, CP 09340, Del. Iztapalapa, CDMX, Mexico; ^6^Medical Research Unit in Biochemistry, Specialties Hospital, National Medical Center SXXI, Instituto Mexicano del Seguro Social, Av. Cuauhtémoc 330, Col. Doctores, Del. Cuauhtémoc, CP 06720, CDMX, Mexico

## Abstract

*Catharanthus roseus *(L.) G. (*C. roseus*) is a medicinal plant used traditionally for diabetes mellitus control. Several compounds of an alkaloidal nature have been proposed as hypoglycemic principles. However, little attention has been paid to other compounds in this plant that could also participate in this hypoglycemic activity. This study aimed to analyze the hypoglycemic effect of a polyphenolic fraction from* C. roseus*, as well as its action on insulin secretion and expression in RINm5F cells.* Methods*. An alkaloid-free aqueous extract was obtained from* C. roseus *stems. The hypoglycemic effect of different doses of this extract was evaluated in normal and streptozotocin-induced diabetic mice. This extract was fractionated by bipartition, and the resultant fractions were assessed by their hypoglycemic effects. Subsequently, the fraction with the greater hypoglycemic activity was added to the RINm5F cells, and the expression and secretion of insulin were analyzed. The antioxidant activity was determined by the DPPH method and through chromatographic analysis of the most active fraction by HPLC, using an Econosphere C18 column.* Results*. The aqueous alkaloid-free extract of* C. roseus* stems significantly reduced blood glucose in normal and diabetic mice. The fractionation of this extract provided three fractions, one of which (a precipitate) showed significant reductions in glycemia at 6 h (48.1 and 64.5% in normal and diabetic mice, respectively). This precipitate contained phenolic compounds and saponins. Its chromatographic analysis showed that it is formed by several phenolic compounds; gallic acid (0.053%) and chlorogenic acid (0.216%) were identified and quantified.* Conclusion*. The phenolic fraction of* C. roseus* containing gallic acid and chlorogenic acid had a hypoglycemic effect that may be explained by an increase in insulin secretion.

## 1. Introduction

Catharanthus* roseus *(L.) G. Don (Syn.* Vinca rosea* L.) (*C. roseus)* is a perennial tropical medicinal plant (Apocynaceae family) used traditionally for diabetes mellitus control [1, 2].* Catharanthus roseus *is one of the best studied medicinal plants, with most of the research focusing mainly on its alkaloids; however, the characterization of other classes of secondary metabolites is scarce [3, 4]. Regarding its use in the treatment of diabetes, a metabolic disease characterized by chronic hyperglycemia with a high prevalence and mortality rate worldwide [5], an alkaloid-free fraction from the aqueous extract of* C. roseus* stems exhibited a hypoglycemic effect in alloxan-induced diabetic mice. The HPLC analysis of this alkaloid-free fraction displayed the presence of at least three unidentified polyphenolic compounds [2]. In this sense, the content of polyphenols in plants is essential because they are natural antioxidants [6], are able to neutralize free radicals, can prevent cellular damage, and offer a protective effect against the development of diabetes complications [7].

In fact, chronic hyperglycemia, the main characteristic of diabetes, is associated with the activation of metabolic pathways that cause continued overproduction of reactive oxygen species (ROS) and an imbalance between antioxidant system activation and ROS production, which results in oxidative stress. Oxidative stress conditions cause DNA fragmentation, oxidation and misfolding of proteins, mitochondrial permeability alteration, and other organelle damage. Patients with type 2 diabetes (T2D) have serum markers of oxidative stress [8]. The alterations, due to the increase of ROS, affect insulin action and secretion by interfering with insulin signaling and ATP production in mitochondrial respiration; in addition, they can activate the intrinsic path of apoptosis and decrease the *β* cell mass [9]. Therefore, ROS increase is linked to insulin resistance, damage and dysfunction of different organs, and the development of diabetic complications such as retinopathy, nephropathy, neuropathy, and vascular injury [10, 11]. Therefore, it is very important to regulate ROS levels in diabetic patients. This can be achieved by controlling hyperglycemia and reinforcing antioxidant defenses. In this regard, it is known that plants are rich in antioxidant compounds and free radical scavengers, such as polyphenols compounds [4]. Therefore, these compounds have been proposed as potential agents for preventing and treating many oxidative stress-related diseases, such as diabetes mellitus [12].

The present research aimed to determine the hypoglycemic effect of a polyphenolic fraction from* C. roseus* and its activity on insulin secretion and expression in RINm5F cells.

## 2. Materials and Methods

### 2.1. Plant Material

*Catharanthus roseus *var.* albus* G. Don, collected in September 2014 in the town of Rio Viejo, Tecuala Municipality, Nayarit, México, was authenticated in the herbarium at Universidad Autónoma Metropolitana (Voucher Specimen 70150 UAMIZ).

#### 2.1.1. Extract Preparation

The stems were dried and ground. The alkaloid-rich extract was obtained using a Soxhlet apparatus with ethanol, and this process was suspended until the ethanol extract did not show alkaloid presence in the Dragendorff test [2]. After this, the mark was subjected to reflux with distilled water for 30 min to obtain an alkaloid-free aqueous extract. The water was eliminated, and the extract was kept at 4°C.

#### 2.1.2. Aqueous Alkaloid-Free Extract Fractionation

The alkaloid-free aqueous extract (5.0 g) was dissolved in distilled water (400 mL). The aqueous phase was filtered and placed in a separation funnel with 200 mL of ethyl acetate. The organic fraction and emulsion were recovered, and the solvent was removed by vacuum evaporation. Subsequently, 200 mL of ethyl acetate was added to the aqueous fraction, and the process was repeated until exhaustive extraction. The water was eliminated via water bath. The emulsion formed between the two fractions was treatment with ethanol to obtain a precipitate. Both portions, aqueous and organic (ethyl acetate), as well as the precipitate, were dried in the extraction hood and stored at 4°C until use.

### 2.2. Animals

Male mice,* Mus musculus *CD-1 strain, weighing 35–45 g, were used. The procedure protocol was approved by Academic Committee of Ethics of the Autonomous Metropolitan University-Iztapalapa. Mice were maintained (5 per cage) according to international rules (NIH guidelines for the handling and care of animals), as well as the Mexican Official Standard (NOM-062-ZOO-1999). The mice were kept under an automatic light and darkness cycle (12* × *12 h) and were maintained at a temperature of 22* ± *1°C. The animals were fed with an elemental diet (Agribrands Purina México, S.A de C.V.) and water* ad libitum*. Animals were fasted for 12 h before the experiment, with access to water only, and were deprived of both food and water during the test.

#### 2.2.1. Hypoglycemic Effect of the Aqueous Alkaloid-Free Extract and Its Fractions in Normal Mice

Mice were separated into 8 groups of 5 mice each and received an intraperitoneal (IP) injection with the following treatments: Group 1 control, saline solution (4 mL/kg); Group 2, glibenclamide as a positive control (10 mg/kg); Groups 3, 4, and 5, different doses of aqueous alkaloid-free extract (125, 250, and 500 mg/kg, respectively); Group 6, aqueous fraction (250 mg/kg); Group 7, organic fraction (250 mg/kg); and Group 8, precipitate (250 mg/kg). Blood samples were collected from the tail vein before (basal glycemia, t=0) and 2, 4, and 6 h after treatments. Glucose quantification was carried out using the glucose oxidase method with an Accu-Chek™ Performa glucometer (Roche Diagnostics, USA).

#### 2.2.2. Hypoglycemic Effect in Streptozotocin-Induced Diabetic Mice after Diabetes Induction

A single IP dose of 135 mg/kg of streptozotocin (STZ) in citrate buffer pH 4.5 was administered. After eight days, blood glucose was measured with an Accu-Chek Performa glucometer (Roche®) by a once-off tail prick. Animals with glycemic levels ≥ 200 mg/dL were considered diabetic and were treated as follows: Group 1 control, saline solution (4 mL/kg); Group 2, glibenclamide as a positive control (10 mg/kg); Group 3, 250 mg/kg of the aqueous alkaloid-free extract; Group 4, aqueous fraction (250 mg/kg); Group 5, organic fraction (250 mg/kg); and Group 6, precipitate (250 mg/kg). Blood samples were collected from tail puncture before (basal glycemia, T=0) and 2, 4, and 6 h after treatments. Glucose concentrations were measured using an Accu-Chek Performa glucometer (Roche®). The results are presented as the percentage of glycemia reduction in relation to the basal glycemia.

#### 2.2.3. Effect of the Fractions on Insulin Secretion in Normal Mice

Two groups of three fasted (8 h) normal mice were anesthetized with an IP injection (100 mg/kg) of xylazine and ketamine (10 mg/kg); then, a heparinized silastic catheter (20 U/mL) was inserted into the left carotid artery. The aqueous fraction and precipitate were injected (250 mg/kg) into the stomach with a needle, and 100 *μ*L of blood was obtained at 0, 15, and 60 min [13]. The blood was centrifuged, and the serum was separated and stored at -20°C until it was assayed. Insulin concentration was measured with an ALPCO™ Rat Insulin ELISA kit, following the supplier's instructions.

### 2.3. RINm5F Cells

RINm5F is an insulin-producing cell line derived from a pancreatic islet tumor acquired from the American Type Culture Collection (ATCC® CRL-11605™). RINm5F cells were cultured in RPMI 1640 medium (GIBCO™) supplemented with 10% fetal bovine serum (SFB), 1 mM pyruvate, 1 mM nonessential amino acids, 2 mM L-glutamine, and 100 U/mL gentamicin in a humid atmosphere at 37°C and 5% CO_2_ until confluence, approximately 72 h [9].

#### 2.3.1. Cellular Viability (MTT)

Cell viability was evaluated by an MTT test, which is based on the ability of cellular dehydrogenases to reduce the bromide of 3-(4,5-dimethylthiazol-2yl)-2,5-diphenyl tetrazolium (MTT) to formazan. Five thousand cells by well were seeded in a 96-well plate (Nunc) with the supplemented RPMI-1640 medium. On the following day, cells were washed with PBS, and the aqueous fractions and the precipitate from the alkaloid-free aqueous extract of* C. roseus* stems (15, 31.25, 62.5, 125, 250, and 500 *μ*g/mL in RPMI 1640 medium without serum) were added. After 24 h, the medium was removed, and the MTT solution (0.1 mg/mL in PBS) was added, incubating for 3 h at 37°C and 95% humidity. The medium was removed and 0.04 M HCl in 2-isopropanol was added for 15 min to dissolve the formazan. The absorbance was registered at 570 nm by an ELISA reader, and the results expressed the viable cell percentage [14, 15].

#### 2.3.2. Expression of Insulin mRNA in RINm5F Cells Treated with the Precipitate from* C. roseus*

RINm5F cells (1x10^6^) were seeded into 6-well plates with the supplemented RPMI medium until confluence. After 24 h, the medium was replaced with a medium containing the* C. roseus* precipitate (10 and 100 *μ*g/mL) and incubated for 1, 2, 4, 8, 16, and 24 h. Cells were then washed with PBS. RNA extraction was performed with Trizol™ following the manufacturer's instructions. The precipitate (total RNA) was dried under vacuum and resuspended in RNase-free water. The concentration and purity of the RNA were determined by spectrophotometry (EPOCH) at A260 and A280 nm, with an A260/A280 ratio of 1.8-2.2 indicating pure RNA, while the RNA integrity was analyzed by agarose gel electrophoresis.

The cDNA was synthesized by retrotranscription using the enzyme M-MuLV-RT (New-England Biolabs™, USA), following the manufacturer's instructions. The reaction was carried out with 1 *μ*g of RNA in a final volume of 20 *μ*L. The reaction mixture contained 4 *μ*M hexamers (Fermentas™, UK), 1X Reverse Transcriptase Buffer 75 mM KCl, 50 mM Tris-HCl, 3 mM MgCl2, 10 mM dithiothreitol (New-England Biolabs™, USA), 10 *μ*M dNTPs (Promega®, USA), 20 U RNase Inhibitor (Ambion™, USA), and 100 U MuLV Reverse Transcriptase (New-England Biolabs™, USA). The reverse transcription reaction was carried out under the following conditions: 42°C for 60 min, 90°C for 10 min, and a final temperature of 4°C/∞.

RT-qPCR was performed using the thermal cycler Mx3005p, MxPro qPCR v.3.00 program (Agilent Technologies™, USA), with the SYBR Green™ detection system. Specific primers were used for insulin and *β*-actin (Table 1). Each reaction was performed in triplicate in a final volume of 20 *μ*L containing the following components: SYBR Green Master Mix 1X (Applied Biosystems™, USA), 0.4 *μ*M sense initiator, 0.4 *μ*M antisense initiator (IDT, USA), nuclease-free water, and 100 ng of cDNA. The amplification was carried out under the following conditions: denaturation of one cycle at 95°C for 10 min, 40 cycles at 95°C for 15 sec, and 56°C for 60 sec. *β*-actin was used as the housekeeping gene. The relative expression was determined by the comparative double Δ^Ct^ method [16].

#### 2.3.3. Insulin Secretion

RINm5F cells (200,000/well) were seeded in 24-well plates with a supplemented RPMI medium and allowed to confluence. The medium was then changed by Ringer-Krebs buffer (115 mM NaCl, 4.7 mM KCl, 1.3 mM CaCl_2_, 1.2 mM KH_2_PO_4_, 1.2 mM MgSO_4_, 24 mM NaHCO_3_, 10 mM HEPES, 1 g/L BSA, and 1.1 mM glucose, pH 7.4) for 2 h to leave the cells in a fasting state. Subsequently, the medium was replaced with another medium that was free of serum, containing the fractions of the aqueous alkaloid-free extract from* C. roseus* (aqueous fraction and precipitate; 10 and 100 *μ*g/mL, respectively) and glibenclamide (4 *μ*M). The medium was collected after 60 min and the insulin concentration was measured with the ALPCO™ Rat Insulin ELISA kit following the supplier's instructions.

### 2.4. Polyphenolic Compound Quantification

The total polyphenolic compounds were measured by the Folin-Ciocalteu method, as previously reported [17]. Gallic acid was used as a standard at different concentrations (2, 4, 6, 8, and 10 *μ*g). Simultaneously, 40 *μ*L of the samples (5 mg/mL) were placed in test tubes. After this, the Folin-Ciocalteu reagent was added (250 *μ*L); 5 min later, a 20 % sodium carbonate solution (1250 *μ*L) was added. Deionized water was then added to bring the volume up to 2.0 mL. After 2 h, the absorbance was measured (UNICO brand™) at 760 nm. The samples' absorbances were compared to the standard curve, and the result was expressed as mg of gallic acid equivalent/100 mg of dry sample. A mixture of water and reagents was used as a blank. This measurement was done in triplicate, and the mean was calculated.

### 2.5. Flavonoid Content Determination

Total flavonoids were measured by the aluminum chloride colorimetric method [18]. An aliquot (100 *μ*L) of extract, precipitate, or fraction (5.0 mg/mL) was mixed with distilled water (125 *μ*L) followed by the addition of a 5.0% sodium nitrite solution (75 *μ*L). After 6 min, 150 *μ*L of the aluminum chloride solution (10%) was added. This mixture was incubated at room temperature (5 min), 500 *μ*L of sodium hydroxide (1 M) was added, and the final volume was completed to 2.5 mL with distilled water. The absorbance of the pink complex was measured at 510 nm using an SQ-2802 spectrophotometer (United Products and Instruments, Inc., USA) and compared to a calibration curve (0.5-10 *μ*g) of catechin (Sigma-Aldrich Chemical, St. Louis MO, USA). Total flavonoids were expressed as mg of catechin equivalents/100 mg of extract, precipitate, or fraction.

### 2.6. Test for Saponins

The presence of saponins was determined using the method of Edeoga [19]. Approximately 20 mg of the sample was boiled in 20 ml of distilled water in a water bath and filtered. Then, 1 ml of the filtrate was mixed with 5 ml of distilled water and shaken vigorously to obtain a stable, persistent foam. The froth was combined with three drops of olive oil and stirred vigorously. Then, the mixture was observed for the formation of an emulsion.

### 2.7. DPPH Assay

The antioxidant activities of the aqueous alkaloid-free extract and its fractions (aqueous, ethyl acetate, and precipitate) were determined using DPPH (1,1-diphenyl-2-picrylhydrazyl). This method was modified from Thaipong [20]. The extract and its fractions were dissolved in a mixture of water-methanol (15:85) at a concentration of 5 mg/mL, and the mixtures were used to prepare different dilutions (ranging from 50 to 500 *μ*g/mL) in methanol. The sample dilution (0.250 mL) was added to 2.25 mL of the DPPH mixed with the new methanol solution (50 *μ*M) to produce the test solutions, while methanol (2.25 mL) was added to 250 *μ*L of the sample as a reference. Another 2.25 mL of the DPPH solution was mixed with methanol (250 *μ*L) to prepare the blank. The solutions were kept in the dark at room temperature for 30 min to let them react. The measurement of absorbance was done at a wavelength of 517 nm. The following equation was applied to convert the absorbance values into a percentage of antioxidant activity:

(1) $$\begin{array}{c} \%\;\;Inhibition=\left[\;\frac{\left(B-T\right)}{B}\;\right]\times 100 \end{array}$$

where B is the absorption of the blank sample and T is the absorption of tested samples.

Linear regression determined the half maximal inhibitory concentration (IC50; *μ*g/mL), and DPPH scavenging activity was determined.

### 2.8. High-Performance Liquid Chromatography (HPLC) Analysis

HPLC analyses were performed on a Waters 600 system with a 2996 diode detector. The analytic separation was performed using an Econosphere C18 column (250 mm, x 4.6 mm, particle size 5 *μ*m). The precipitate (2 mg/mL) was filtered through a 0.2 *μ*m filter. A volume of 20 *μ*L of sample was injected. The solvents used for gradient elution were acetonitrile and acidified water (pH 2.5) in varying proportions (0 min, 0:100; 10 min, 15:85; 15 min, 20:80; and 30 min, 0:100). The total run time was 30 min at 0.8 mL/min, and the oven temperature was set to 35°C. Gallic acid (0.07 mg/ml), quercetin, chlorogenic acid, ferulic acid, rutin, and protocatechuic acid (0.1 mg/ml of each) standards were used to compare the absorption spectra and retention times. Experiments were done in duplicate, and results are reported as a percentage (mg of gallic acid or chlorogenic acid / 100 mg sample). The contents of gallic acid and chlorogenic acid in the precipitate were calculated using the area values of sample and standar, as well as the standard concentration.

### 2.9. Statistical Analysis

Data are presented as the mean and standard error of the mean (S.E.M.). In vitro experiments were performed in triplicate in independent events. In all cases, the significant differences between treatments were determined by an analysis of variance (ANOVA) followed by a Tukey-Kramer multiple-comparison test (*p *< 0.05).

## 3. Results

### 3.1. Hypoglycemic Effect of the Alkaloid-Free Extract and Fractions from* C. roseus* Stems in Normal Mice

The effect of different doses of the aqueous alkaloid-free extract and glibenclamide on the glycemia of normal mice is shown in Table 2. The data are presented as the percentage of glucose reduction versus basal glycemia. At doses of 125 mg/kg, this extract decreased blood glucose at 4 and 6 h (*p *< 0.05), while the hypoglycemic effect of the 250 mg/kg dose began after 2 h (*p *< 0.05) and was maintained throughout the experiment. The dose of 500 mg/kg reduced glycemia at 4 h and remained unchanged for 6 h. Therefore, the concentration of 250 mg/kg was chosen as the concentration to be used in the experiments with the fractions in normal and streptozotocin-induced diabetic mice. Although the three fractions obtained from the alkaloid-free extract, the aqueous fraction, the organic fraction, and the precipitate, showed an increase in blood glucose at 2 h after administration (Table 2), they also caused significant reductions of glycemia at 6 h (*p* < 0.05).

### 3.2. Hypoglycemic Effect of the Aqueous Alkaloid-Free Extract and Fractions from* C. roseus* Stems in Diabetic Mice

The impacts of the aqueous alkaloid-free extract of* C. roseus*, saline solution, and glibenclamide on glycemia in mice with experimental diabetes are shown in Table 3. The data are presented as the percentage of reduction of fasting blood glucose. The alkaloid-free aqueous extract significantly reduced glycemia (*p* < 0.05), similar to glibenclamide at 2 h, and maintained the reduced glycemia throughout the experiment. The aqueous fraction and the precipitate exerted a hypoglycemic effect for 4 h, with a reduction in blood glucose of 51% and 33.79 %, respectively, and maintained a reduction for 6 h, with a decrease of 58.60 % and 64.06 %, respectively. The organic fraction did not present a hypoglycemic effect.

### 3.3. Effect of the Aqueous Fraction and the Precipitate from* C. roseus* on Insulin Secretion in Normal Mice

**Figure 1:**
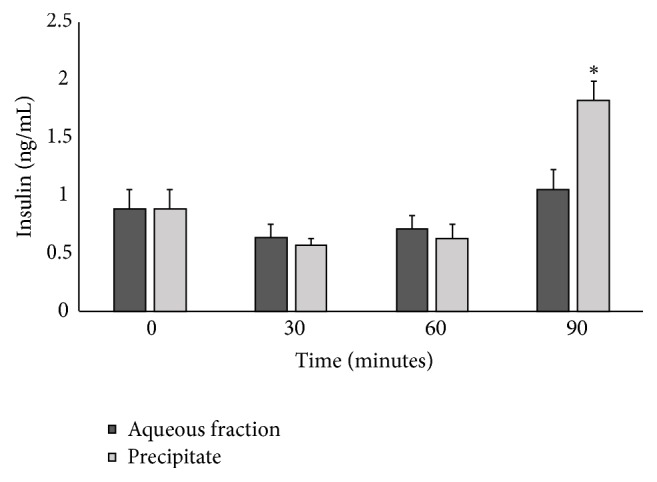
Insulin concentration in normal mice treated with the aqueous fraction and precipitate of the aqueous alkaloid-free extract of* C. roseus*. n=3. Significantly different from basal glucose *∗*p < 0.01.

The impact of the aqueous fraction and the precipitate from* C. roseus* on insulin secretion in normal mice is shown in Figure 1. The fractions increased insulin secretion by 35.6 % and 135.6 % at 90 min, but the increase was significant only for the precipitate (*p* < 0.05).

### 3.4. Effect of the Aqueous Fraction and the Precipitate from* C. roseus* Stems on Cellular Viability

**Figure 2:**
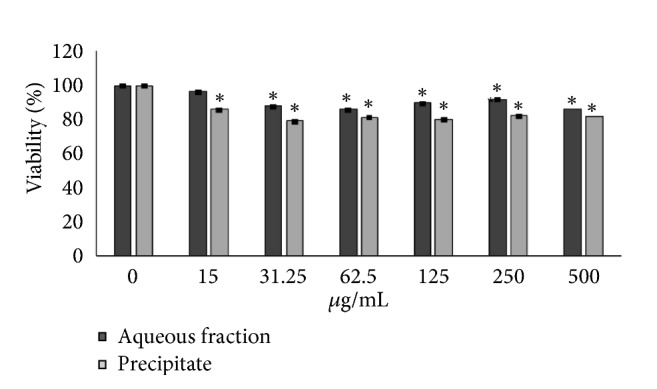
Effect of the aqueous fraction and the precipitate of the aqueous alkaloid-free extract of* C. roseus* on the viability of RINm5F cells (MTT assay, T= 24 h). n= 9. *∗* means significantly different versus control, p < 0.05.

Figure 2 shows the viability percentages of RINm5F cells treated with different concentrations of the aqueous fraction and the precipitate of* C. roseus*. None of the doses showed cytotoxic effects, but the cell viability decreased in a dose-dependent manner versus the control group (*p* < 0.05). Therefore, to continue with the subsequent experiments, the concentrations chosen were 10 and 100 *μ*g/mL for both treatments.

### 3.5. Effect of the Aqueous Fraction and the Precipitate from* C. roseus* Stems on Insulin Expression in RINm5F Cells

**Figure 3:**
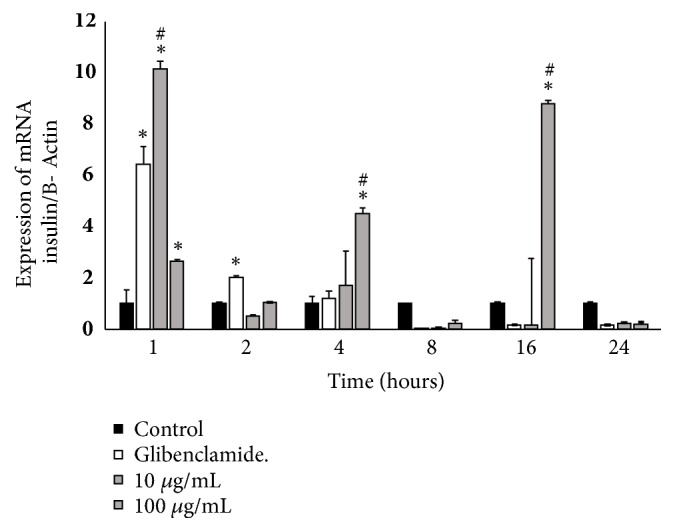
Insulin mRNA expression in RINm5F cells treated with precipitate obtained from the aqueous alkaloid-free extract of* C. roseus*, n =3. *∗*, ^#^ mean significantly different versus control or glibenclamide at the same time. n= 3.* p *< 0.01.

With both fractions, a reduction in the expression of insulin mRNA (concerning *β*-actin) was observed (Figure 3). At concentrations of 10 and 100 *μ*g/mL, the aqueous fraction reduced mRNA expression by 15 and 37 %, while the precipitate decreased mRNA expression by 39.6 and 30.9 %, respectively (*p* < 0.01).

### 3.6. Effect of the Aqueous Fraction and the Precipitate from* C. roseus* Stems on Insulin Secretion in RINm5F Cells

**Figure 4:**
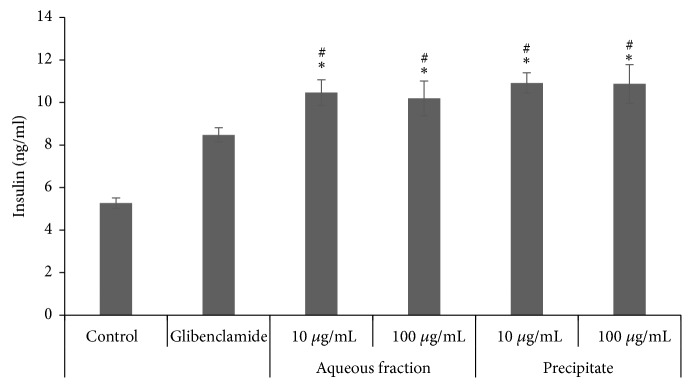
Effect of the aqueous fraction and the precipitate obtained from the alkaloid-free aqueous extract of* C. roseus* on insulin secretion. *∗*, ^#^ mean significantly different versus control or glibenclamide. n= 3.* p* < 0.05.

Figure 4 shows the impact of* C. roseus* fractions on insulin secretion in RINm5F cells cultured with 5.5 mM glucose. It was observed that the aqueous fraction and the precipitate stimulated insulin secretion by approximately 50 %, with respect to the control. There were no significant differences between the concentrations of the extracts (10 and 100 *μ*g/mL). As expected, glibenclamide increased insulin secretion by 37.7 % versus the control (*p *< 0.05).

### 3.7. Chemical Analysis

The content of total polyphenols (mg gallic acid equivalents/100 mg of sample) and flavonoids (mg catechin equivalents/100 mg of sample) and the antioxidant activity (CI50, *μ*g/mL), of the aqueous alkaloid-free extract from* C. roseus* and its fractions are shown in Table 4. The aqueous alkaloid-free extract contains 6.33 mg (mg gallic acid equivalents/100 mg of sample) total polyphenols, whereas its fractions contained 1.86, 3.6, and 1.2 mg (mgGAE/100 mg of sample) total polyphenols for the aqueous fraction, organic fraction, and precipitate, respectively. This result suggests that there are other types of compounds in the alkaloid-free extract's chemical composition, such as saponins, because this extract was positive for the foam test with a length of 1.7 cm, and the olive oil test corroborated this result.

Likewise, the aqueous alkaloid-free extract contains 2.37 mg CTE/100 mg of sample (flavonoids) and the fractions contain 0.67 % (aqueous fraction), 1.52 mg CTE/100 mg of sample (organic fraction), and 0.6 mg CTE/100 mg of sample (precipitate) flavonoids.

The free radical scavenging activities was measured as the sample necessary to decrease the initial concentration of DPPH by 50 % (IC50, *μ*g/mL). The IC 50 values for the alkaloid-free aqueous extract, aqueous fraction, organic fraction and precipitate were 120.51, 181.08, 140.36 and 300.15, respectively.

### 3.8. Chromatographic Analysis of the Precipitate of the Alkaloid-Free Aqueous Extract from the* C. roseus* Stem

Chromatographic separation showed several peaks. The absorption spectra of retention times 14.19 and 20.15 min, are shown in Table 5. It was observed that the compound eluted to 14.19 min corresponds to gallic acid and the compound eluted to 20.15 min correspond to chlorogenic acid. The gallic acid and chlorogenic acid are present in the precipitate in amounts of 0.053 % and 0.216 %, respectively.

## 4. Discussion

The use of plants to treat various diseases is a practice that has been increasing recently. However, few plants scientifically validated and, therefore, their safety and efficacy are unknown [21].* Catharanthus roseus* is one of the medicinal plants used worldwide; an infusion of its stem, leaves, and flowers is used to treat diabetes [1, 2, 22]. Scientific reports have shown that an ethanolic extract from the leaves of* C. roseus* decreases glycemia levels in healthy and diabetic rats [23–25].

In this research, we showed that the aqueous alkaloid-free extract from* C. roseus* stems decreased glycemia in normal and diabetic mice, as previously reported by Vega-Avila [2]. All tested fractions (aqueous fraction, organic fraction, and the precipitate) from this extract also presented a hypoglycemic effect in normal mice with a reduction of glycemia of up to 22, 31, and 48 %, respectively. However, in diabetic mice, the best impacts were seen with the aqueous fraction and the precipitate, which reduced blood glucose by 58 and 64 %, respectively; therefore, these fractions were used to continue the chemical characterization and* in vitro* studies.

In the present study, a liquid-liquid fractionation was made, resulting in an aqueous fraction, an organic fraction, and a precipitate. The precipitate was analyzed by HPLC, and the chromatogram showed several compounds, of which two were identified. Some phenolic compounds have been identified in some parts of* C. roseus*, including vanillic acid, gallic acid, ferulic acid, chlorogenic acid, kaempferol, and quercetin [4, 26]. It is known that phenolic compounds can counteract oxidative stress generated during chronic hyperglycemia. The long-term elevation of blood glucose levels is associated with macro- and microvascular complications due to the increase in oxidative stress, in which free radicals have a main role in the pathogenesis [27, 28]. In this sense, the American Diabetes Association (ADA) [10] recommended the consumption of antioxidants, such as polyphenols, to counteract the oxidative stress generated during hyperglycemia and to delay the onset of diabetic complications. Phenolic compounds act as reducing agents, hydrogen donors, singlet oxygen quenchers, or metal chelators. Therefore, the content of polyphenols in antidiabetic plants is essential, because they prevent cellular damage and protect against the development of diabetes complications [7, 29]. Additionally, it has been reported that phenolic compounds impact carbohydrate metabolism through various mechanisms, such as inhibiting digestive enzymes, delaying intestinal glucose absorption, and stimulating insulin secretion by pancreatic *β*-cells [30]. In this study, the chemical analysis indicated that the aqueous alkaloid-free extract and its fractions contain polyphenols. Phenolic compounds include several groups such as phenolic acid (gallic acid and chlorogenic acid) and flavonoids [26], and we quantified the gallic acid (0.053 %) and chlorogenic acid (0.216 %) by HPLC. The gallic acid, also known as 3,4,5-trihydroxybenzoic acid, is a phenolic acid present in various natural sources, and it has different biological effects, such as anti-inflammatory, antioxidant, antimicrobial, cardiovascular protective, and anticancer properties [31]. Gallic acid regulates various biological processes, including the reduction of the oxidation of low-density lipoproteins (LDL) that carry cholesterol in the blood, thus preventing diseases such as atherosclerosis [32].

To determine if insulin secretion mediates the* in vivo* hypoglycemic effect of the precipitate, an* in vitro* study was carried out in RINm5F cells. For this study, an MTT assay was first carried out with different concentrations of the precipitate. None of the doses substantially affected the cellular viability, even at doses as high as 500 *μ*g/mL. This result supports the hypothesis that the precipitate of the alkaloid-free fraction of* C. roseus* had no cytotoxic effect.

The precipitate increased the secretion of insulin in RINm5F cells; however, we did not observe any effect on the mRNA expression of insulin. Since the same result was obtained with glibenclamide, it is possible to suggest that the precipitate acts as a secretagogue of insulin in a similar way that glibenclamide does, by closing the K^+  ATP^ channels, depolarizing the plasmatic membrane cell, increasing the concentrations of intracellular Ca^2+^, and inducing the secretion of insulin in RINm5F cells. This secretagogue action can be generated by the gallic acid present in the precipitate because it has been reported that gallic acid increases insulin secretion in a dose-dependent manner in RINm5F cells and protects them from glucolipotoxicity due to its antiapoptotic actions [32].

Chlorogenic acid (CGA) is one of the most abundant polyphenol compounds in the diet, especially in coffee. It is an ester formed from cinnamic acids and quinic acid and is also known as 5-O-caffeoylquinic acid (5-CQA) [33]. It has been shown that CGA exhibits many biological properties, including antibacterial, antioxidant, and anticarcinogenic activities. Concerning its effects on hyperglycemia and hypertriglyceridemia, it is known that chlorogenic acid inhibits *α*-glucosidase, thus reducing postprandial hyperglycemia. CGA also inhibits Na^+^ dependent glucose transport and increases the expression of GLUT4; this effect is comparable to metformin [30]. The precipitate decreased glycemia and increased insulin secretion in normal mice. Chlorogenic acid could cause this effect since it has been described as an insulin secretagogue by increasing the levels of intracellular Ca^2+^. It also acts as an insulin sensitizer, increasing the levels of PPAR*γ*, PPAR*α*, and GLUT4 [34]. Our results indicate that the precipitate possesses many compounds with different mechanisms of action; they could be beneficial for the development of therapeutic agents for the treatment of type 2 diabetes.

## 5. Conclusions

The precipitate obtained from the aqueous alkaloid-free extract of* C. roseus* contains gallic acid and chlorogenic acid and has a hypoglycemic effect in normal and diabetic mice. This effect is due, at least in part, to the stimulation of insulin secretion.

## Figures and Tables

**Table 1:** Sequence of insulin primers and *β*-actin.

Gen	Sense	Antisense
INS-2	5′-GGAGCGTGGATTCTTCTACAC-3′	5′-CAGTGCCAAGGTCTGAAGG-3′
*β*-actin	5′-TTCCATCCTCCAGAAACCAG-3′	5′-CCCTCGAACTAAGGGGAAAG-3′

**Table 2:** Effect of the aqueous alkaloid-free extract of *C. roseus* and its fractions in normal mice.

**Treatment **	**Dose **	**Blood glucose reduction (**%**)**		
		2 h	4 h	6 h
Saline solution	4 mL/kg	5.8 ± 2.55	6.6 ± 1.25	-5.7 ± 1.49
Glibenclamide	10 mg/kg	14.9 ± 2.11*∗*	32.6 ± 0.57*∗*	1.7 ± 2.06*∗*
Aqueous alkaloid-free extract	125 mg/kg	0.8 ± 1.18	13.0 ± 1.51*∗*	23.5 ± 1.60*∗*
	250 mg/kg	17.9 ± 3.85*∗*	35.3 ± 2.65*∗*	25.0 ± 8.29*∗*
	500 mg/kg	3.7 ± 4.04	46.0 ± 1.6*∗*	53.2 ± 1.5*∗*
Aqueous fraction	250 mg/kg	-26.3 ± 7.83	25.6 ± 6.15*∗*	22.3 ± 5.58*∗*
Organic fraction	250 mg/kg	-29.2 ± 5.53	1.3 ± 5.68	31.6 ± 5.64*∗*
Precipitate	250 mg/kg	-33.5 ± 6.2	7.2 ± 3.35	48.1 ± 1.55*∗*

Values are the mean percentage of blood glucose reduction (±S.E.M.). *∗* indicates significant differences compared with fasting glycemia (*p* < 0.05), n=5.

**Table 3:** Blood glucose reduction (%) in diabetic mice due to the administration of the aqueous alkaloid-free extract and its fractions obtained from *Catharanthus roseus*.

**Treatment **	**Dose **	**Blood glucose reduction (**%**)**		
		2 h	4 h	6 h
Saline solution	4 mL/kg	12.7 ± 7.64	15.0 ± 6.42	12.5 ± 6.61
Glibenclamide	10 mg/kg	24.0 ± 4.68*∗*	37.0 ± 3.61*∗*	43.5 ± 5.98*∗*
Aqueous alkaloid-free extract	250 mg/kg	26.5 ± 7.61*∗*	28.2 ± 7.94*∗*	40.6 ± 6.2*∗*
Aqueous fraction	250 mg/kg	1.2 ± 6.27	49.2 ± 7.26*∗*	51.2 ± 19.3*∗*
Organic fraction	250 mg/kg	-22.2 ± 4.19	-0.09 ± 6.32	2.9 ± 9.35
Precipitate	250 mg/kg	2.6 ± 9.35	35.2 ± 4.48*∗*	64.5 ± 1.71*∗*

Values are the mean percentage of blood glucose reduction (±S.E.M.). *∗* indicates significant differences compared with fasting glycemia (*p* < 0.05), n=5.

**Table 4:** Quantification of total polyphenols, total flavonoids and antioxidant activity (IC50) of aqueous alkaloid-free extract and its fractions.

**Extract/fraction**	**Polyphenols (**%**)**	**Flavonoids (**%**)**	**Antioxidant activity CI50 (** ***μ*** **g/mL)**
Aqueous alkaloid-free extract	6.33	2.37	120.54
Aqueous fraction	1.86	0.67	181.08
Organic fraction	3.60	1.52	140.36
Precipitate	1.20	0.67	300.15

Polyphenols (%) based on mgGAE/100 mg of sample. Flavonoids (%) based on mgCTE/100 mg.

**Table 5:** Spectral analysis and quantification of compounds in the precipitate from *C. roseus*.

Compound	Retention time (min)	% in the sample	Spectral signal (sample)	Spectral signal (standard)
Gallic acid	14.19	0.053	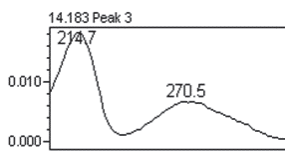	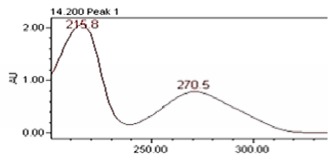
Chlorogenic acid	20.15	0.216	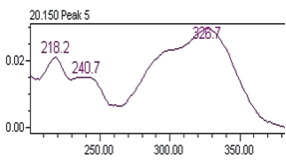	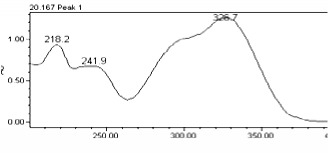

## Data Availability

The data used to support the findings of this study are included within the article.
